# Measurement of Water Holdup in Vertical Upward Oil–Water Two-Phase Flow Pipes Using a Helical Capacitance Sensor

**DOI:** 10.3390/s22020690

**Published:** 2022-01-17

**Authors:** Runsong Dai, Ningde Jin, Qingyang Hao, Weikai Ren, Lusheng Zhai

**Affiliations:** School of Electrical and Information Engineering, Tianjin University, Tianjin 300072, China; drs97@tju.edu.cn (R.D.); QGHA@nnit.com (Q.H.); renweikai1222@tju.edu.cn (W.R.); lszhai@tju.edu.cn (L.Z.)

**Keywords:** oil–water two-phase flow, helical capacitance sensor, water holdup measurement, high water cut

## Abstract

Oil–water two-phase flows widely exist in industrial production, especially in the petroleum industry. The liquid holdup is significant for understanding reservoir production characteristics and improving oil recovery. This paper focuses on the helical capacitance sensor for the measurement of water holdup of oil–water two-phase flows. A new double helix capacitance sensor with an electrode rotation angle of 360° is designed. The sensitivity field distribution of the sensor with different parameters is simulated by the finite element analysis method, and the optimal geometric size of the sensor is obtained. The measurement characteristics of the sensor under different flow conditions are investigated by dynamical experiments of vertical oil–water flows. By analyzing the response signal of the helical capacitance sensor, the flow pattern can be identified, and the apparent water holdup can be calculated. The results show that the proposed sensor is suitable to measure the water holdup in a wide range of water cuts. Even in flow conditions of a high water cut, the sensor still retains good resolution in the D O/W flow pattern. This study expands the water holdup measurement of a capacitance sensor in the case of an oil–water two-phase flow with a high water cut.

## 1. Introduction

Oil–water two-phase flow is very common in natural and industrial processing. It is very difficult to detect the parameters of oil–water two-phase flow due to the difference of inherent properties between phases, such as the fluid density and viscosity. Liquid holdup measurement of oil–water two-phase flow is significant to understand reservoir production characteristics and improve oil recovery. 

After long-term exploitation, many traditional oilfields will enter the stage of high water cut exploitation. Due to the large amount of formation water and long-term water injection production, the average water cut of the oilfield may become excessive, and the phenomenon of low production and low permeability is very serious [1,2,3,4,5,6]. How to measure the liquid holdup of two-phase flow under the conditions of a high water cut has attracted increasing attention in the international community.

There is slippage between the oil phase and water phase, which leads to the complexity of local concentration distribution and velocity distribution of mixed fluid. According to the flow relationship between the water phase and oil phase, six common flow patterns of oil–water two-phase flow have been defined [7,8], in which the oil in water flow pattern when water is in the continuous phase is divided into the dispersion of oil in water flow (D O/W), very fine dispersion of oil in water flow (VFD O/W) and oil in water churn flow (O/W CF). When the oil is the continuous phase, the flow pattern can be divided into water in oil churn flow (W/O CF), dispersion water in oil flow (D W/O) and very fine dispersion water in oil flow (VFD W/O). Common two-phase flow measurement methods include the conductivity method [9,10], capacitance method [11,12], optical method [13,14], ultrasonic measurement method [15,16], etc. The electrical sensors have the advantages of high sensitivity, a stable signal and convenient use. Compared with conductivity sensors, capacitance sensors are less affected by fluid conductivity and are widely used in the field of multiphase flow measurement.

In the development of a two-phase flow sensor, capacitance sensors have been studied and explored for a long time. Abouelwafa et al. [17] divided capacitance sensors into six categories, including parallel plates, concave plates, staggered concave plates, double helix, multiple helix and four concave plates capacitance sensor. They proposed that the capacitive sensors with four concave plates or double helix structure have the best detection sensitivity. 

Geraets et al. [18] made a detailed study on capacitive sensors with helical structure and expounded the necessity of protective electrodes. They considered the pipe section thickness also has impact on the measurement results. Hammer et al. [19] pointed out that the double helix capacitance sensor had little dependence on the change of flow pattern. Experiments have verified that it has a good measurement performance on the uniform mixed flow of water in crude oil. 

Tollefsen and Hammer [20] designed a capacitance sensor based on 180° helical electrode and found that it has better robustness and can effectively reduce flow pattern dependence. Elkow et al. [21] designed helical and concave electrode capacitance sensors to measure the void fraction of vertical gas–liquid two-phase flow in small tubes. It was found that the geometry of helical capacitance sensor has a great influence on the linearity of its response.

In order to solve the problem of realtime measurement of two-phase flow in the industrial injection pipeline, Jin Feng et al. [22] designed a helical capacitance phase concentration measurement sensor for gas–solid two-phase flow. Jaworek et al. [23] proposed a concave capacitance sensor for phase holdup measurement, that measures the capacitance of mixed fluid through LC resonant circuit and designed eight channels to measure different positions of the pipeline. Hu Hongli et al. [24] developed a helical capacitance sensor measurement system and applied it to the measurement of gas–solid two-phase flow in the pneumatic powder conveying pipeline of boiler to ensure the stability of material combustion. 

Hu Jinhai et al. [25] designed a composite capacitive sensor composed of a cylindrical capacitor and coaxial capacitor and carried out electric field simulation and optimization, which has greatly improved the measurement effect compared with the traditional coaxial capacitive sensor. Ye Jiamin et al. [26] proposed a helical capacitance sensor for measuring gas–liquid two-phase flow in a small diameter and optimized the structure of the sensor by finite element simulation.

Emerson dos Reis et al. [27] conducted a detailed analysis of a parallel plate capacitance sensor, concave plate capacitance sensor, double ring capacitance sensor and double helix capacitance sensor, and compared these sensors through experiments. They installed the above sensors in the same horizontal pipe section and matched the horizontal gas–liquid two-phase laminar flow under different flow conditions. They found that the double ring capacitive sensor had the best measurement characteristics for gas–liquid two-phase flow. 

Zhao An et al. [28] optimized the structure of a wall capacitance sensor through simulation, studied its holdup measurement characteristics for oil–water two-phase flow in horizontal pipeline. Zhai Lusheng et al. [29] designed a capacitance sensor based on a 180° double helix electrode for oil–water two-phase flow under the conditions of a horizontal pipeline and measured the response of the sensor.

In summary, the capacitive sensor has a rich structure, and the research of helical capacitance sensors has attracted extensive attention. Due to its independence to the fluid flow structure and sensitive measurement characteristics, it has significant advantages in two-phase flow detection. In this paper, a helical capacitance sensor is applied to the research of vertical rising oil–water two-phase flow. 

A helical capacitance sensor with a rotation angle of 360° is designed. On this basis, other sensor parameters are simulated and optimized. The sensor is designed and manufactured. Finally, the corresponding experiments are conducted, and the results are analyzed.

## 2. The Principle of Helical Capacitance Sensor

### 2.1. Sensor Structure

**Figure 1:**
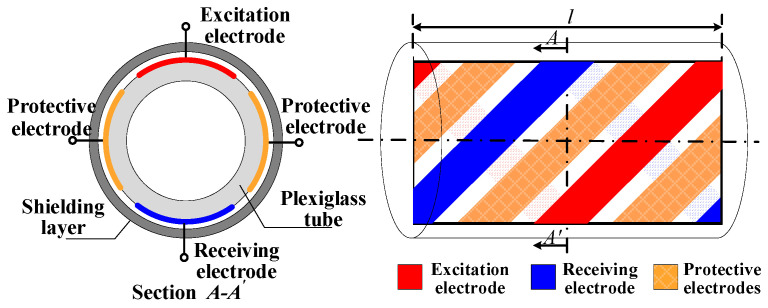
Structure of a double helix capacitance sensor.

As shown in Figure 1, the electrode structure of the double helix capacitance sensor can be divided into measuring electrodes, protective electrodes and a metal shielding layer. The measuring electrodes and protective electrodes are arranged on the outer wall of the plexiglass tube and rotated 360° along the pipe axial direction. The metal shielding layer is sleeved on the outside of the measuring electrodes and protective electrodes to shield the divergent electric field. When the mixed liquid flows through the measurement pipeline, due to the difference in the dielectric constant between oil and water, the capacitance of the mixed fluid will change with the change of the water holdup. Therefore, the water holdup can be calculated by measuring the capacitance of the mixed fluid and combining the corresponding models.

### 2.2. Sensor Detection Principle

The helical capacitance sensor forms a detection electrostatic field in the vertical plexiglass tube. The potential distribution in the electric field is described by a Poisson equation as follows [30]:

(1) $$\nabla^{2}\phi =-\frac{\rho }{\varepsilon }$$

where ϕ is the potential, *ρ* is the charge concentration and ε is the dielectric constant. For the electric field in three-dimensional space, ignoring the influence of charge distribution, the potential distribution in the field can be written as:

(2) ∇⋅[ε(x,y,z)∇φ(x,y,z)]=0

where x,y,z∈Ω, Ω represents the electric field area; ε(x,y,z) is the dielectric constant distribution of the field, ϕ(x,y,z) is the point potential of grid nodes [20]. After meshing, the potential distribution in the field can be written as the integration of each part,

(3) $$F(\phi )=\underset{\Omega }{∭}\left\{\frac{1}{2}\varepsilon \left[{\left(\frac{\partial \phi }{\partial x}\right)}^{2}+{\left(\frac{\partial \phi }{\partial y}\right)}^{2}+{\left(\frac{\partial \phi }{\partial z}\right)}^{2}\right]\right\}dxdydz=\sum_{e=1}^{m}F_{e}(\phi )$$

F(ϕ) represents the electric field energy of the whole area, $F_{e}\left(\phi \right)$ represents the electric field energy at the subdivision units, *M* is the total number of divided units. According to the above formula, the point potential of all grid nodes, ϕ(x,y,z), can be obtained, and thus the electric field intensity $\vec{E}$ can be calculated:

(4) $$\vec{E}=-\nabla \phi$$

Select a closed surface containing the measuring electrodes and accumulate the node potential previously obtained by the finite element method on this surface to obtain the charge of the sensor measuring electrodes, which can be written as:

(5) $$C=\frac{Q}{U}$$

*U* represents the potential difference between the two electrodes, *Q* is the amount of charge, and *C* is the capacitance value. Through the above calculation process, the simulated capacitance between the electrodes can be obtained.

Hammer and Tollefsen et al. [19] analyzed the equivalent capacitance corresponding to different capacitor plate orientations under the condition of oil-water stratified flow. According to the theory of Hammer and Tollefsen et al., if the plate direction is perpendicular to the stratified flow, the capacitance measured by the sensor, *C_V_*, can be regarded as the parallel connection of two capacitors:

(6) $$C_{V}=\varepsilon_{0}\varepsilon_{oil}\left(1-Y_{w}\right)+\varepsilon_{0}\varepsilon_{w}Y_{w}$$

where $Y_{w}$ is the water holdup,$\varepsilon_{0}$ is the dielectric constant of vacuum, $\varepsilon_{oil}$ is the relative permittivity of the oil and $\varepsilon_{w}$ is the relative permittivity of water. When the plate direction is parallel to the stratified flow, the measured capacitance value, *C_H_*, can be regarded as the series connection of two capacitors:

(7) $$C_{H}=\frac{\varepsilon_{0}\varepsilon_{oil}\varepsilon_{w}}{\varepsilon_{w}+\left(\varepsilon_{oil}-\varepsilon_{w}\right)Y_{w}}$$

It is obvious that there is a linear relationship between *C_V_* and water holdup $Y_{w}$, while the relationship between *C_H_* and $Y_{w}$ is hyperbolic. When $Y_{w}$ is low, the change of *C_H_* with $Y_{w}$ is slow. With the increase of $Y_{w}$, the growth rate of *C_H_* with $Y_{w}$ is faster and faster. However, the oil–water two-phase flow fluid structure is complex and cannot be simply regarded as the parallel or series connection of a resistor and a capacitor but the series and parallel connection of many resistors and capacitors. According to the experimental results of Liu Xingbin et al. [31], the response of a capacitive sensor with a water cut is more similar to an S-shaped curve. When the water cut is very high or very low, the response of the capacitance sensor changes slowly with the water cut.

## 3. Optimization of Sensor Structure Parameters

Finite element analysis (FEA) has been widely used in capacitor electrode design. Xie et al. [30] systematically studied the sensitivity distribution of concave capacitance sensors based on a two-dimensional finite element model and indicated that the wall thickness of a dielectric tube is one of the important parameters affecting the uniformity of the sensitivity field. There is an optimal value for measuring the electrode angle. 

Jin et al. [32] proved that the protective electrodes in the double helix capacitance sensor can improve the sensitivity of the central measurement area, and the tube wall thickness has little effect on the uniformity of the sensitivity field. Canière et al. [33] considered the influence of flow pattern on sensor design and optimized the concave capacitance sensor for a small diameter tube. 

Cao and Wang [34] constructed a geometric optimization mathematical model of a helical capacitance sensor for gas–solid two-phase flow measurement. The designed sensor can produce an almost uniform sensitivity field, which provides good linear characteristics for gas–solid two-phase flow concentration measurement.

**Figure 2:**
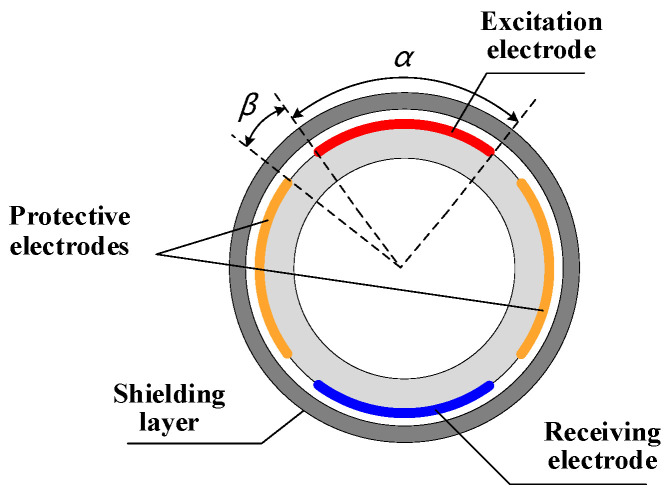
Schematic diagram of a sensor pipe section.

Figure 2 is the cross-sectional view of the pipe where the helical capacitance sensor is located. The cross-section angle of the measuring electrodes, *α*, and the interval angles between the measuring electrodes and the protective electrodes, *β*, are important parameters affecting the measurement characteristics of the sensor. In addition, the electrode pitch length, *l* (as shown in Figure 1), is also an important structural parameter of the sensor. When the above parameters change, the sensitivity distribution of the capacitance sensor will change significantly. Therefore, in this chapter, the parameters are simulated and optimized to obtain the optimal structure of the sensor.

### 3.1. Optimization Principle of Sensor Simulation

For a helical capacitance sensor, the change of fluid in the measurement pipeline will affect the capacitance of the measurement area. The change of capacitance can be reflected by the sensitivity of the sensor. The sensitivity calculation formula of capacitive sensor is defined as

(8) $$S(k)=\frac{C(\varepsilon_{k})-C(\varepsilon_{o})}{C(\varepsilon_{w})-C(\varepsilon_{o})}\;k=1,2,3....M$$

where $C\left(\varepsilon_{w}\right)$ is the capacitance value when the fluid medium in the pipeline is all water, $C\left(\varepsilon_{o}\right)$ is the capacitance value when the medium is all oil, $C\left(\varepsilon_{k}\right)$ is the capacitance value when the unit *k* is set as water and the rest is set as oil, and *M* represents the total number of division units. The dielectric constants of water and oil are set to 80 and 2.5, respectively. The simulation unit type is 3D Brick. 

Set different partition units as water phase in turn and simulate the change of capacitance in the measurement space. Compare this with the simulation results that the measurement space is all oil to obtain the sensitivity at the location of the partition unit. Then, investigate the sensitivity distribution of the whole sensor measurement space. 

In this way, the sensitivity distribution of the whole sensor measurement space can be investigated. In this paper, the average sensitivity (*S_avg_*) and the sensitivity variation parameter (SVP) proposed by Xie et al. [30] are used as the evaluation indicators of the sensitivity field. It is expected that the final structure has a high average sensitivity and low uniformity error. The *S_avg_* calculation formula of the helical capacitance sensor is:

(9) $$S_{avg}=\frac{1}{M}\sum_{j=1}^{M}S_{j}$$

The SVP is defined as

(10) $$\mathrm{SVP}=\frac{S_{dev}}{S_{avg}}\times 100\%$$

where *S_dev_* is the sensitivity standard deviation of divided units, which can be calculated by the following formula:

(11) $$S_{dev}={\left[\frac{1}{M}\sum_{j=1}^{M}{\left(S_{j}-S_{avg}\right)}^{2}\right]}^{1/2}$$

It can be seen from the above analysis that the larger *S_avg_*, the higher the detection sensitivity of the capacitive sensor, and the smaller SVP is, the more uniform the sensitivity distribution in the measurement space. The purpose of the optimization process is to find the parameter values for maximum *S_avg_* and minimum SVP.

### 3.2. Optimization of the Angle between Excitation and Protection Electrodes

**Figure 3:**
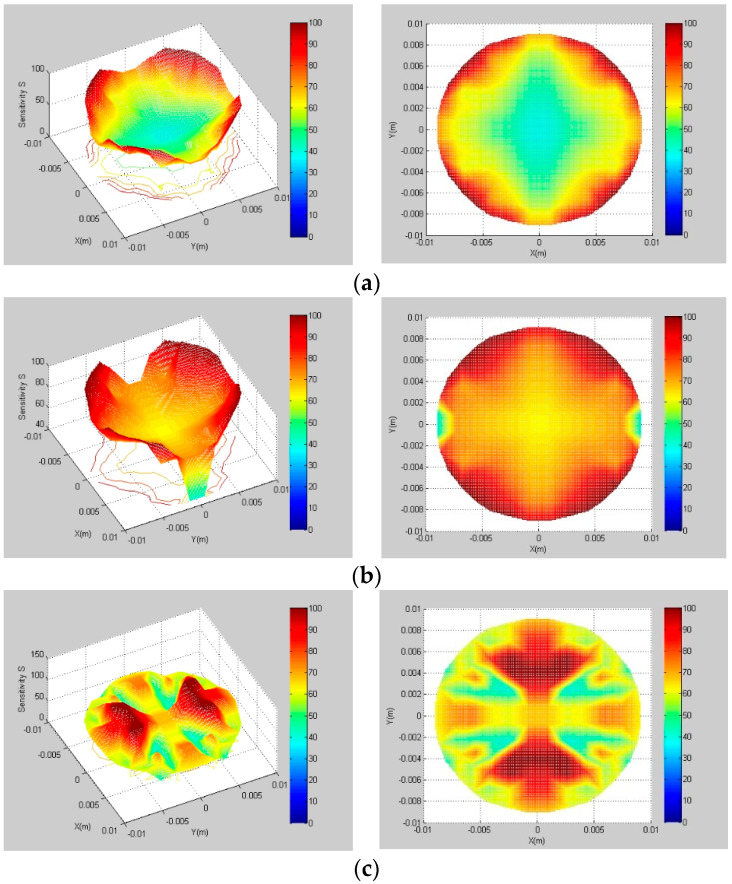
Sensitivity field with different interval angles, β. (**a**) β=25°; (**b**) β=15°; (**c**) β=5°.

According to experience, for optimizing the angle between the measuring and protective electrodes, the measuring electrode angle and the pitch length are set as 60° and 94 mm, respectively. When the interval angle, β, is 30°, 25°, 20°, 15°, 10° and 5°, the sensitivity field of the helical capacitance sensor pipe section was studied. Part of the results are shown in the Figure 3.

The sensitivity field indexes under different interval l angles are calculated, and the results are shown in Table 1. It can be found from Figure 3 and Table 1 that, when the interval angle is 15°, the SVP is the smallest, and the *S_avg_* is the largest. Therefore, 15° is selected as the best interval angle of the helical capacitance sensor.

### 3.3. Optimization of Measuring Electrode Opening Angle

**Figure 4:**
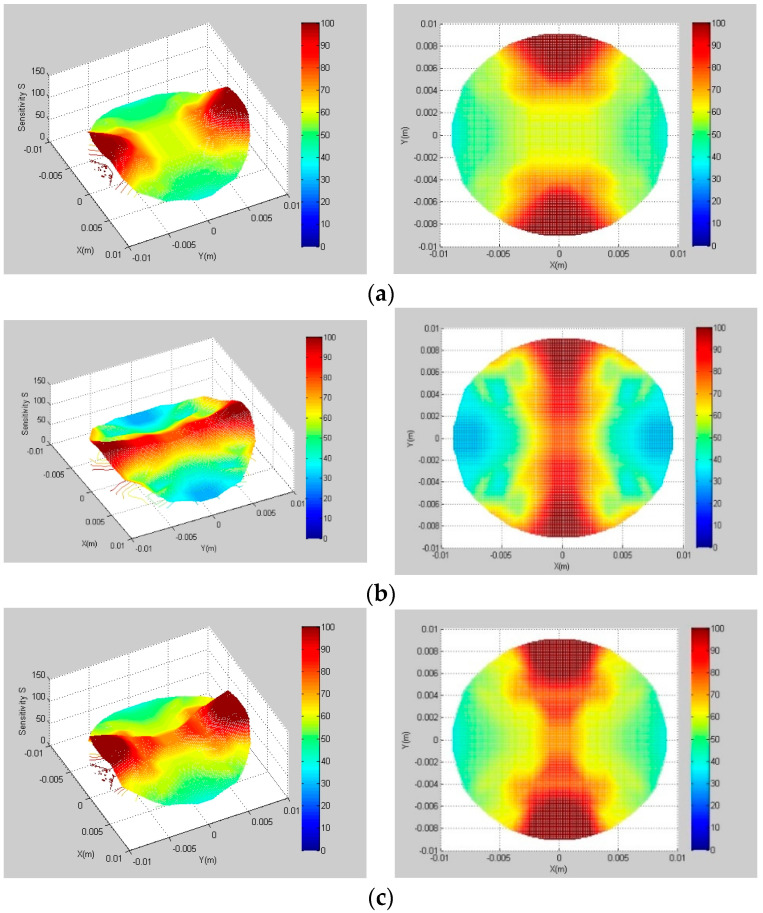
Sensitivity field with different opening angles of measuring electrodes, *α.* (**a**) α=62°; (**b**) α=66°; (**c**) α=70°.

The interval angle between the measuring and protective electrodes is set to 15°, and the pitch length remains 94 mm when optimizing the opening angle of the measuring electrodes, α. According to the previous optimization experience, the optimization range is set from 60° to 70°. Parts of the simulation results are shown in Figure 4.

The sensitivity field indexes with different opening angles, *α*, are calculated. The results are shown in Table 2. It can be found from Figure 4 and Table 2 that, when the measuring electrode opening angle is 66°, the SVP is the smallest, and the *S_avg_* is the largest. Therefore, 66° is selected as the optimal measuring electrode opening angle of the sensor. According to the measuring electrode opening angle, *α*, and interval angle, *β*, it can be determined that the opening angle of the protection electrode is 84°.

### 3.4. Optimization of Electrode Pitch

**Figure 5:**
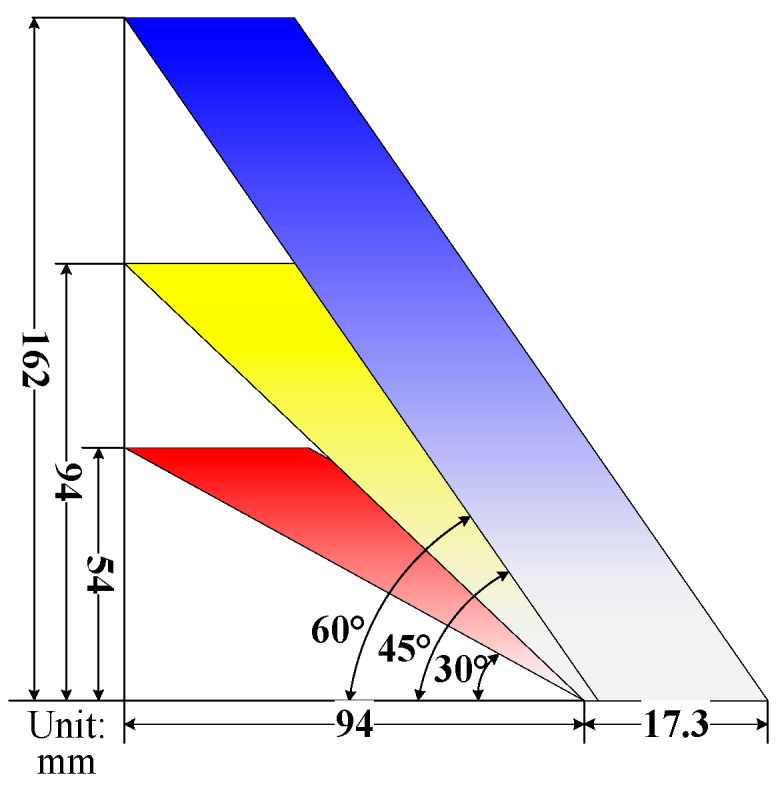
Schematic diagram of expanded electrodes.

The optimization of the sensor electrode pitch will affect the sensitivity field distribution in three-dimensional space. The concept of the electrode winding angle is introduced in this paper. The pitch length of the electrode helical structure is determined by the initial deflection angle of horizontal winding when the electrode rotates along the tube wall. If the outer diameter of the pipe is 30 mm and the electrode rotates 360°, there must be an expanded electrode with a horizontal displacement of 94 mm, as shown in Figure 5. When the corresponding electrode winding angle is 30°, 45° or 60°, the required pitch is 54, 94 or 162 mm, respectively.

**Figure 6:**
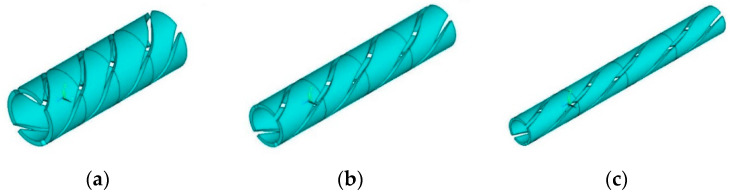
Schematic diagram of electrodes with different pitches. (**a**) *l* = 54 mm; (**b**) *l* = 94 mm; (**c**) *l* = 162 mm.

The winding angles are set to 30°, 45° and 60°, and the helical structure of the measuring electrodes and the protective electrodes are shown in Figure 6.

**Figure 7:**
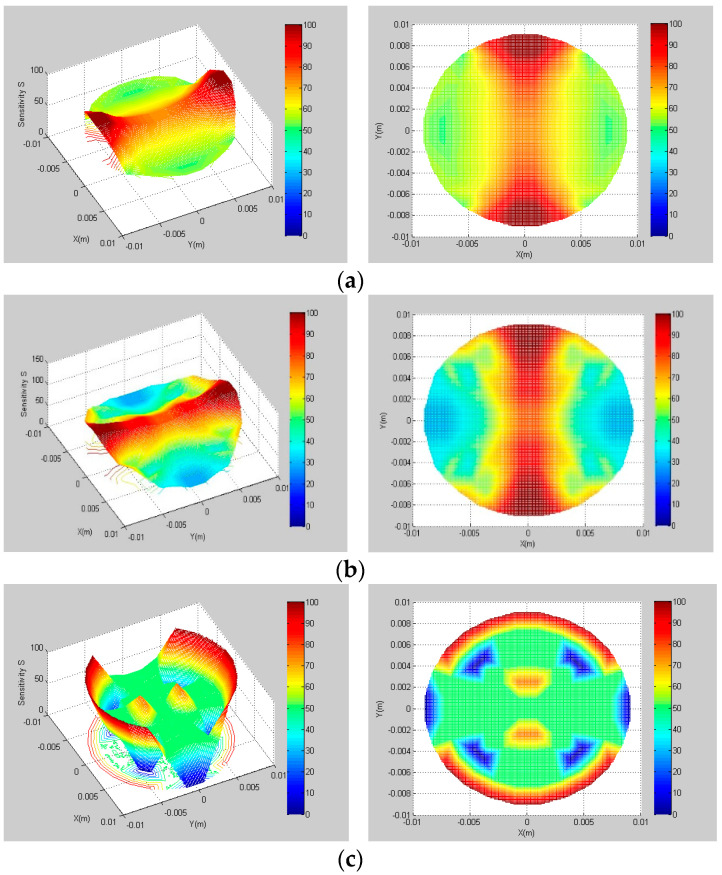
Sensitivity fields with different pitches. (**a**) l=54 mm; (**b**) l=94 mm; (**c**) l=162 mm.

The sensitivity fields of sensor section corresponding to different pitches are shown in Figure 7. When the winding angle is 30° with *l* = 54 mm, the overall sensitivity of the pipe section is higher, including the area near the protective electrodes. The reason is that the sensor pitch is small, which makes the rotation angle of the electrode larger in the same length of the pipe, and thus the overall measurement sensitivity is high. When the winding angle is 45° with *l* = 94 mm, the sensitivity near the protective electrodes is weakened. When the winding angle increases to 60° with *l* = 162 mm, the sensitivity field will change greatly. The original focusing and deep detection ability of the sensor will disappear with the significant increase of pitch. Only the area near the measuring electrodes has good sensitivity; other areas are very poor.

It can be found from Table 3 that the measurement characteristics of the sensor are similar when the winding angle is 30° and 45°. However, in the actual oil–water two-phase flow experiment, the length of the longest oil slug is about 80 mm. In order to make the sensor pipe completely contain the oil slugs during the measurement, 94 mm is selected as the sensor pitch.

Finally, the optimal parameters of the sensor are determined as follows: the angle between the section measuring electrode and the protective electrode is 15°, the angle of the section measuring electrode is 66°, the winding angle of the electrode is 45°, and the pitch of the sensor *l* = 94 mm.

## 4. Vertical Upward Oil–Water Two-Phase Flow Experiment

### 4.1. Sensor and Measuring Circuit

**Figure 8:**
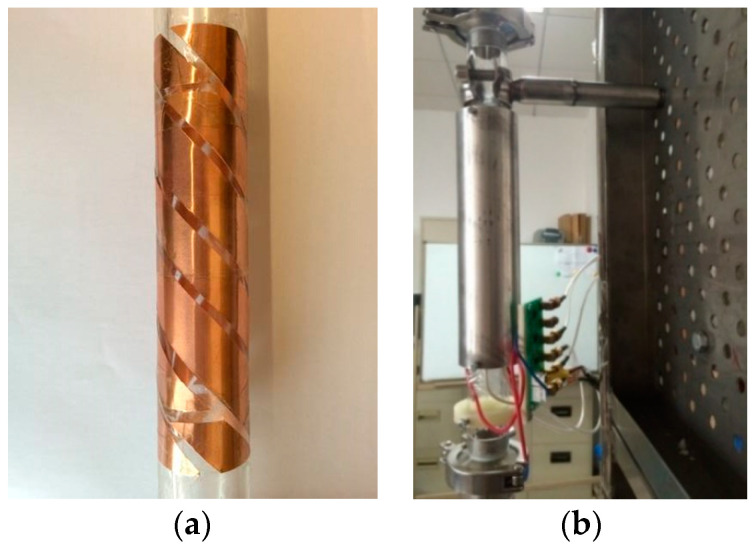
Double helix capacitance sensor: (**a**) without shielding layer; (**b**) with shielding layer and measuring circuit.

According to the previous simulation results, the final parameters of the sensor were determined. The actual double helix capacitance sensor is shown in Figure 8. In order to reduce the interference of external noise signal on the measurement signal of the sensor, an aluminum shielding layer was sleeved outside the capacitive sensor. Figure 8a is the sensor without shielding layer, and Figure 8b is the sensor with a shielding layer and partial measuring circuit.

**Figure 9:**
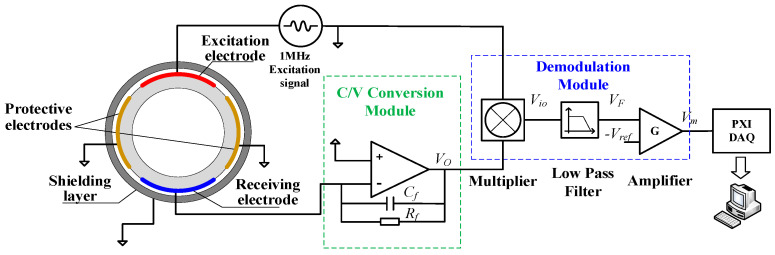
Measurement system diagram of a helical capacitance sensor.

AC excitation is selected as the excitation mode of the capacitance sensor, which can effectively reduce the interference of the distributed capacitance near the measured area. The measuring circuit is composed of a signal generator, C/V conversion module, programmable gain amplification module, signal demodulation module, signal low-pass filter module, signal differential amplification module, A/D module and single-chip microcomputer, as shown in Figure 9. In order to reduce the influence of empty tube capacitance, DC compensation is adopted.

The expression of sinusoidal excitation signal is $V_{I}=A\sin(\omega t+a)$, *A* is the amplitude of signal, *a* is the initial phase of the signal, and ω is the signal angular frequency. According to the study of Marco Demori et al. [35], when the excitation signal frequency is greater than 1 MHz, the dynamic response effect of the sensor is better. Therefore, the frequency of the excitation signal in this paper is 1 MHz. Assume the capacitance of the pipe section to be measured is *C_x_*. Under the excitation of sinusoidal signal, the induced current generated by helical capacitance sensor passes through the operational amplifier and C/V conversion module composed of comparison resistance and comparison capacitor. The expression of the output, *V_o_*, is

(12) $$V_{O}=-\frac{j\omega R_{f}C_{x}}{1+j\omega R_{f}C_{f}}V_{I}$$

Through the multiplier, LPF circuit and amplifier, the relationship between the output voltage of the signal conditioning circuit, *V_m_*, and the capacitance of the helical capacitance sensor, *C_x_*, can be expressed as

(13) $$V_{m}=-K\frac{A^{2}}{2C_{f}}C_{x}+KV_{ref}$$

where *K* is the gain of the amplifier, and *V_ref_*, is the reference voltage of operational amplifier. It can be seen that the relationship between *V_m_* and *C_x_* is linear, and the measurement sensitivity of the signal is $K\frac{A^{2}}{2C_{f}}$. The derivation process is shown in Zhai’s work [29]. The *PXI4472* signal acquisition card of *NI* company was selected to implement the data acquisition. The sampling frequency was 4 kHz. The data collected entered the upper computer and was saved by the LabVIEW program.

### 4.2. Experiment Facility

**Figure 10:**
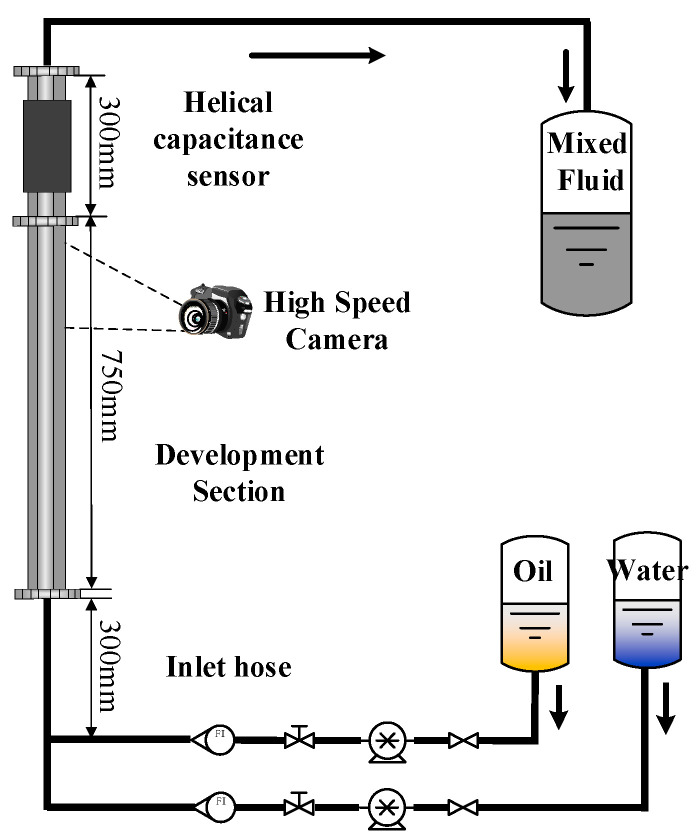
Schematic diagram of a device for a two-phase flow dynamic experiment.

In this paper, the vertical oil–water two-phase flow experiment was performed on a multiphase flow experimental device. The measurement characteristics of a double helix capacitance sensor were investigated. Figure 10 is the schematic diagram of a two-phase flow experimental device.

In the experiment, the plexiglass tube with inner diameter of 20 mm was used as the measuring pipe section. The height of the experimental tube was about 105 cm, and the helical capacitance sensor was placed 75 cm away from the inlet of the mixed liquid to ensure that the mixed liquid has been fully mixed and developed when the mixed fluid flows through the measuring pipe. The high-speed camera under the capacitance sensor was used to capture the flow state of the mixed fluid with an interval of 7.5 ms per frame.

White oil, with a viscosity of 11.984 mPa·s and density of 856 kg/m^3^, and water, with a density of 1000 kg/m^3^ and viscosity of 1 mPa·s, were used in the experiment. The oil–water split phase flow metering device was two WG600F peristaltic pumps.

### 4.3. Experimental Condition Settings

**Figure 11:**
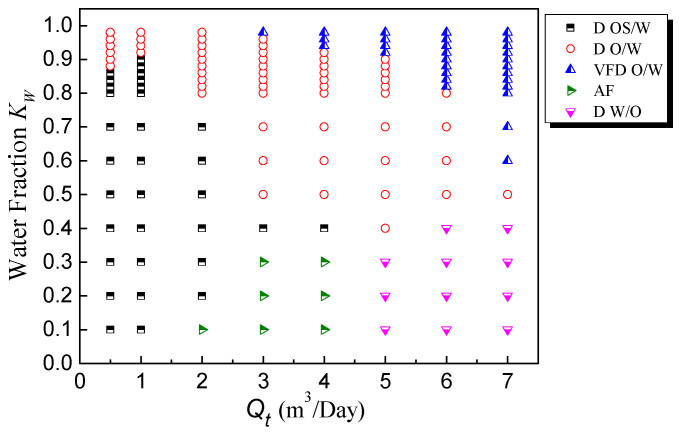
Experimental flow pattern diagram of a vertical oil–water two-phase flow.

In this experiment, the water cut (*K_w_*), the ratio of inlet volumetric flow rate of water over the total inlet volumetric flow rate *Q_t_*, changed from 10% to 98%, covering almost the entire range. There were five flow patterns in the experiments: dispersion oil in water slug flow (D OS/W), annular flow (AF), dispersion water in oil flow (D W/O), dispersion oil in water bubble flow (D O/W) and very fine dispersion oil in water flow (VFD O/W). According to the actual fluid flow structure, the flow patterns under different working conditions were identified. The final flow pattern distribution diagram is shown in Figure 11.

**Figure 12:**
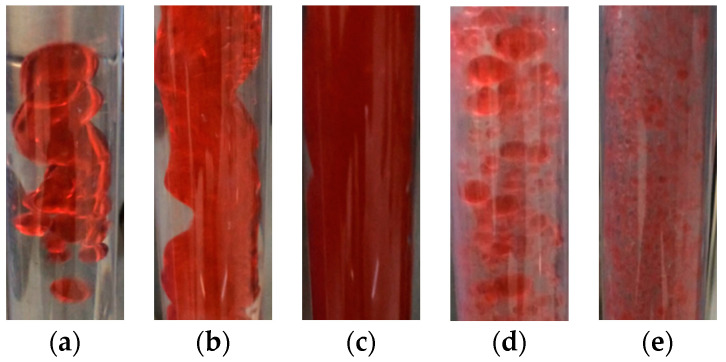
Typical experimental flow pattern structure: (**a**) D OS/W; (**b**) AF; (**c**) D W/O; (**d**) D O/W; (**e**) VFD O/W.

In the experiment, when both the total flow rate (*Q_t_*) and water cut (*K_w_*) were low, the oil bubbles moved slowly and easily coalesced and most of the flow conditions were dispersion oil in water slug flow (D OS/W) (Figure 12a, *Q_t_* = 0.5 m^3^/Day, *K_w_* = 30%). The length of the oil slugs ranged from 5 to 80 mm, and the large oil slugs were often followed by small oil bubbles. 

When the total flow rate was high but the water cut was low, the flow pattern was AF (Figure 12b, *Q_t_* = 4 m^3^/Day, *K_w_* = 20%) or D W/O (Figure 12c, *Q_t_* = 7 m^3^/Day, *K_w_* = 40%). As the water cut increased, the flow pattern became D O/W (Figure 12d, *Q_t_* = 4 m^3^/Day, *K_w_* = 70%). In the high water cut area with a high total flow rate, there were only small oil bubbles in the pipeline (Figure 12e, *Q_t_* = 7 m^3^/Day, *K_w_* = 90%), which were approximately homogeneously dispersed.

## 5. Analysis of Experimental Results

### 5.1. Response Characteristics of Helical Capacitance Sensor

**Figure 13:**
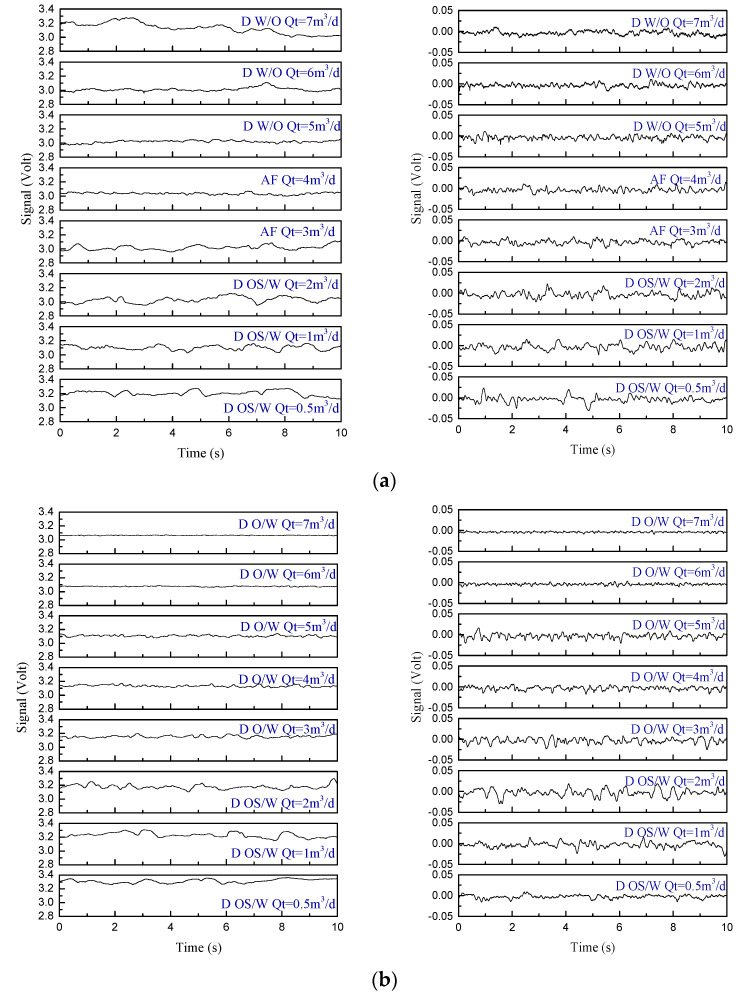

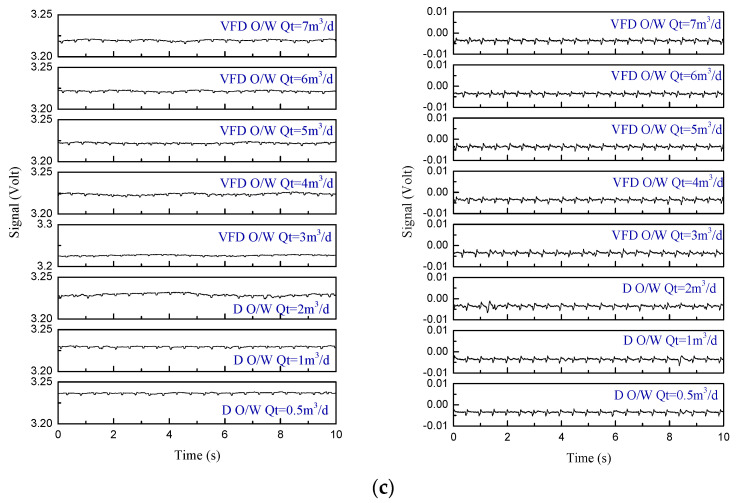
DC (**left**) and AC (**right**) measurement signals under different experimental flow conditions. (**a**) *K_w_* = 20%; (**b**) *K_w_* = 50%; (**c**) *K_w_* = 98%.

In the oil–water two-phase flow experiment, the capacitive sensor adopted both DC and AC excitation modes. The output signals under all flow conditions were measured respectively. When the water cut was 100%, the measured DC voltage, *V_w_*, was 2.8 V. When the water cut was 0%, the measured DC voltage, *V_o_*, was 3.4 V. The AC signal fluctuated within the range of ±0.05 V. This paper shows the measurement signals of flow conditions, as shown in Figure 13. In each group of waveforms, the left side is the DC signal and the right side is the AC signal.

First, the DC voltage signals of the helical capacitance sensor were analyzed. The DC voltage signal fluctuated around a certain value, and the fluctuation of the signal was generally small. The higher the water cut, the higher the reference voltage value of the signal. For D OS/W and D W/O, there were intermittent signal mutations in the signal, indicating that there were large oil slugs or water droplets flowing, resulting in large changes in the water holdup in the measurement area. AF is the transition pattern between oil in a water slug flow and oil in an oil flow. The signal was similar, but the fluctuation was smaller. As the total flow rate and water cut increased, oil droplets were evenly distributed in D O/W and VFD O/W, and the signal showed approximately random high-frequency small-amplitude fluctuations.

For the AC signal, as shown in Figure 13a, it was under the flow condition with a water cut of 20%. The signal of D OS/W showed a fluctuating signal with intermittent signal mutations, indicating that there were large oil slugs flow through the measuring pipe. With the increase of the total flow rate, AF appeared. The voltage signal showed the transition state from D OS/W to D O/W, including both a mutation signal and high-frequency small amplitude fluctuation signal. When the total flow rate continued to increase, the signal of D W/O showed approximately random fluctuations, accompanied by insignificant fluctuation. The reason is that there are large water droplets in the continuous oil phase, resulting in the fluctuation of the sensor measurement signal.

Figure 13b shows the signals of flow conditions with a water cut of 50%. With the increase of the water cut, D O/W appeared. There were continuous large fluctuations in the output signal, which was due to the continuous flow of uneven oil bubbles in the measuring pipe section of the sensor. When the water cut was 98%, there were VFD O/Ws, as shown in Figure 13c. There were only high-frequency fluctuations with small amplitudes in their signals, which corresponds to the flow phenomenon of small oil bubbles approximately random distributed in the pipeline.

### 5.2. Water Holdup Measurement Results

**Figure 14:**
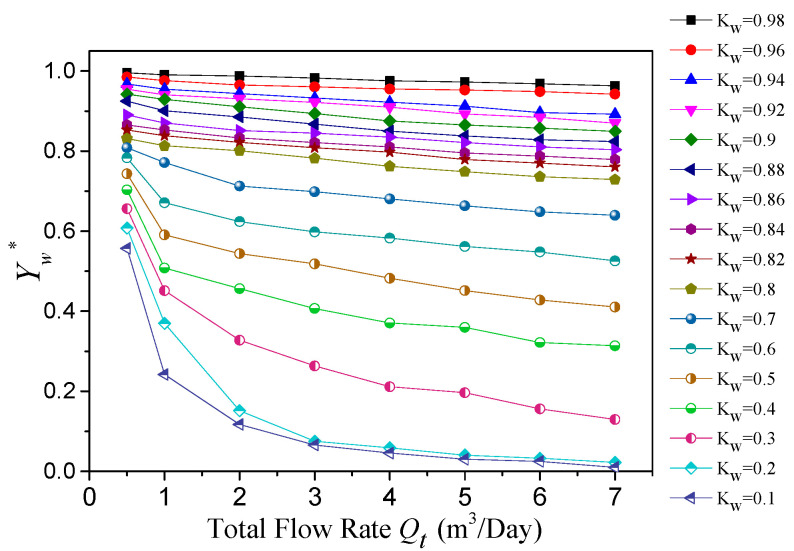
The relationship between the apparent water holdup and water cut.

In this paper, a new parameter apparent water holdup, Y_w_^*^, is defined, and the normalized apparent water holdup under all working conditions is calculated. The calculation formula of normalized apparent water holdup is as follows:

(14) $$Y_{w}^{*}=\frac{V_{m}-V_{o}}{V_{w}-V_{o}}$$

where *V_m_* represents the output DC voltage. *V_w_* and *V_o,_* respectively, represent the DC voltage output when the fluid in the pipeline is all water phase and all oil phase. According to Equation (14), the apparent water holdup chart of the helical capacitance sensor can be calculated, as shown in Figure 14. We found that, in the range of a 10% to 98% water cut, the apparent water holdup measured by the helical capacitance sensor had good resolution. In addition, the sensor showed independence to the flow pattern, which could improve the measurement stability of the sensor.

In Figure 14, the relationship between the apparent water holdup Y_w_^*^, water cut *K_w_* and total flow rate *Q_t_* is analyzed. When the water cut *K_w_* was low, the flow patterns were D OS/W, AF and D W/O. Within this water cut range, the helical capacitance sensor had good measurement performance. In the case of a high oil content, due to the oil phase not being conductive, traditional conductivity sensors will be invalid; however, the capacitance sensor is not affected by the flow pattern.

When the water cut was high, the flow pattern was mainly D O/W and VFD O/W. The proportion of dispersed oil phase was very small, especially VFD O/W. The influence of small oil droplets flowing through the measurement pipeline was very limited, which makes it difficult to measure and distinguish effectively. However, the sensor proposed in this paper still had good resolution under this condition.

When the water cut was less than 20%, the resolution of the sensor was poor. At this time, the flow pattern was mostly oil in water slug flow (D OS/W) or annular flow (AF) with large oil slugs. At this time, the sensor equivalent circuit was more similar to capacitors in series. The variation law of capacitance with water holdup is more similar to the curve of *C_H_* in Equation (7). When the water holdup was low, the capacitance changed slowly with the water holdup, and thus the resolution of the sensor was poor.

In the left part of Figure 14, the total flow rate is less than 1 m^3^/day, the flow pattern was mainly D OS/W. At this time, the phenomenon of small oil bubbles coalescing into large oil bubbles or even oil slugs was very serious. The slippage effect is obvious. The frequency of the dispersed oil phase flowing through the sensor measurement space was very high. Therefore, there is a large difference between the calculated apparent water holdup and actual water cut.

When the water cut was fixed and the total flow rate of mixed fluid increased, the apparent water holdup measured by a helical capacitance sensor tended to a fixed value. As the oil–water two-phase slippage occurs when the total flow is low, and the oil phase velocity in the fluid was greater than the water phase velocity, the apparent water holdup must be greater than the water cut. However, with the increase of the total flow rate of mixed fluid, the influence of slippage is weakened, and the apparent water holdup obtained by the helical capacitance sensor is greatly reduced and finally tends to be stable.

## 6. Conclusions

The parameters of a 360° helical electrode capacitance sensor were simulated and optimized by ANSYS finite element analysis. The measuring electrode cross-section angle, the interval angle between the measuring electrodes and the protective electrodes as well as the helical electrode pitch length of the sensor were studied. Based on the *S_avg_* and SVP of the sensitivity field, the optimal geometry of the sensor was determined. According to the simulation results, a double helix capacitance sensor was designed. 

The dynamical experiments of oil–water two-phase flow were performed, and the responses of the capacitance sensor under the different flow conditions were obtained. The experimental results show that the sensor could distinguish oil–water two-phase flows with different water cuts. Even under the flow conditions of a high water cut, the sensor still had good resolution in the D O/W flow pattern. When the water cut was very low, the measurement characteristics of the helical capacitance sensor were not ideal, and these need to be improved in further work. In conclusion, this study proposed a complete design process for a helical capacitance sensor and expanded the water holdup measurement of a capacitance sensor in the case of an oil–water two-phase flow with a high water cut.

## Figures and Tables

**Table 1:** Sensitivity field evaluation index value with different *β*.

Interval Angle (β)	5°	10°	15°	20°	25°	30°
$S_{avg}$	56.21%	54.55%	**68.41%**	54.71%	55.89%	56.69%
SVP	0.2581	0.2939	**0.1766**	0.2772	0.2628	0.2613

**Table 2:** Sensitivity field evaluation index values with different measuring electrode opening angles.

Measuring Electrode Section Angle (α)	60°	62°	64°	66°	68°	70°
$S_{avg}$	68.41%	65.68%	67.06%	**69.52%**	65.96%	67.53%
SVP	0.1766	0.1646	0.1655	**0.1612**	0.1704	0.1696

**Table 3:** Sensitivity field index with different helical electrode pitches (*l*).

Helical Electrode Pitch (mm)	54	94	162
$S_{avg}$	73.64%	**69.52%**	52.77%
SVP	0.2071	**0.1612**	0.4881

## Data Availability

Not applicable.

## Nomenclature

***Symbol***	
*C*	Capacitance value (F)
*C_x_*	Capacitance of the helical capacitance sensor (F)
*C_V_*	Capacitance of the parallel connection of two capacitors (F)
*C_H_*	Capacitance of the series connection of two capacitors (F)
*C(* $\varepsilon_{k}$ *)*	Capacitance value when the fluid medium is oil water mixture (F)
*C(* $\varepsilon_{o}$ *)*	Capacitance value when the fluid medium is all oil (F)
*C(* $\varepsilon_{w}$ *)*	Capacitance value when the fluid medium in the pipeline is all water (F)
E	Electric field intensity (V/m)
F(ϕ)	Electric field energy of the whole sensor measurement area (J)
$F_{e}\left(\phi \right)$	Electric field energy at the subdivision units (J)
*l*	Pitch length of the electrodes of the capacitance sensor (mm)
*M*	Total number of division units
*Q*	Charge quantity (C)
*Q_t_*	Total flow rate of oil–water flow (m^3^/day)
*S_avg_*	The average sensitivity of the electric field of sensor
*S_dev_*	Sensitivity standard deviation of divided units
*U*	Potential difference between two plates of capacitive sensor (V)
$V_{i}$	Excitation signal of the sensor (V)
$V_{o}$	Output of the C/V conversion module (V)
*V_m_*	Output voltage of the signal conditioning circuit (V)
*V_o_*	Output when the fluid in the pipeline is all oil (V)
*V_w_*	Output when the fluid in the pipeline is all water (V)
*Y_w_*	Water holdup
*Y_w_^*^*	Apparent water holdup
***Greek letters***	
*α*	Cross-section angle of the measuring electrodes (°)
*β*	Interval angles between the measuring electrodes and the protective electrodes (°)
ε	Dielectric constant (F/m)
$\varepsilon_{0}$	Dielectric constant of vacuum
$\varepsilon_{oil}$	Relative dielectric constant of oil
$\varepsilon_{w}$	Relative dielectric constant of water
*ρ*	Charge density (C/m^2^)
ϕ	Electric field potential (V)
***Acronyms***	
SVP	Sensitivity variation parameter
FEA	Finite element analysis
AF	Annular flow
D O/W	Dispersion oil in water flow
D OS/W	Dispersion oil in water slug flow
D W/O	Dispersion water in oil flow
VFD O/W	Very fine dispersion oil in water flow
